# Pharmacokinetics and pharmacodynamics of artesunate and dihydroartemisinin following oral treatment in pregnant women with asymptomatic *Plasmodium falciparum *infections in Kinshasa DRC

**DOI:** 10.1186/1475-2875-10-49

**Published:** 2011-02-28

**Authors:** Marie A Onyamboko, Steven R Meshnick, Lawrence Fleckenstein, Matthew A Koch, Joseph Atibu, Victor Lokomba, Macaya Douoguih, Jennifer Hemingway-Foday, David Wesche, Robert W Ryder, Carl Bose, Linda L Wright, Antoinette K Tshefu, Edmund V Capparelli

**Affiliations:** 1 Kinshasa School of Public Health, Kinshasa, The Democratic Republic of Congo; 2 UNC Gillings School of Global Public Health Department of Epidemiology, 3301 MHRCChapel Hill NC 27599-7435, USA; 3 University of Iowa, Iowa City, Iowa, USA; 4 RTI International, Research Triangle Park, NC, USA; 5 Crucell Holland B.V., Leiden The Netherlands; 6 David Wesche Consulting LLC, Ann Arbor, MI, USA; 7 University of California, San Diego, CA, USA; 8 University of North Carolina at Chapel Hill, NC, USA; 9 National Institute of Child Health and Human Development, NIH, Rockville, MD, USA

## Abstract

**Background:**

In many malaria-endemic countries, increasing resistance may soon compromise the efficacy of sulphadoxine-pyrimethamine (SP) for intermittent preventative treatment (IPT) of malaria in pregnancy. Artemisinin-based IPT regimens represent a promising potential alternative to SP. Pharmacokinetic and safety data supporting the use of artemisinin derivatives in pregnancy are urgently needed.

**Methods:**

Subjects included pregnant women with asymptomatic falciparum parasitaemia between 22-26 weeks (n = 13) or 32-36 weeks gestation (n = 13), the same women at three months postpartum, and 25 non-pregnant parasitaemic controls. All subjects received 200 mg orally administered AS. Plasma total and free levels of AS and its active metabolite DHA were determined using a validated LC-MS method. Non-compartmental pharmacokinetic analysis was performed using standard methods.

**Results:**

All pregnant women delivered live babies. The median birth weight was 3025 grams [range 2130, 3620]; 2 of 26 babies had birth weights less than 2500 grams. Rates of parasite clearance by 12 hours post-dose were high and comparable among the groups. Rapid elimination of AS was observed in all three groups. The 90% CI for the pregnancy:postpartum ratio of geometric means for total and free AUC fell within the pre-specified 0.66 - 1.50 therapeutic equivalence interval. However, more pronounced pharmacokinetic differences were observed between the pregnancy and control subjects, with the 90% CI for the pregnancy:control ratio of geometric means for both total 0.68 (90% CI 0.57-0.81) and free AUC 0.78 (90% CI 0.63-0.95) not fully contained within the 0.66 - 1.50 interval. All subjects cleared parasites rapidly, and there was no difference in the percentage of women who were parasitaemic 12 hours after dosing.

**Conclusions:**

A single dose of orally administered AS was found to be both effective and without adverse effects in this study of second and third trimester pregnant women in the DRC. Although DHA AUC during pregnancy and postpartum were similar, the AUC for the pregnant group was less than the non-pregnant controls. The findings of this study suggest that additional studies on the pharmacokinetics of AS in pregnant women are needed.

**Trial Registration:**

ClinicalTrials.gov: NCT00538382

## Background

Pregnant women are particularly susceptible to malaria, which can lead to intrauterine growth restriction, low birth weight, prematurity, and other poor birth outcomes [1-7]. In order to protect pregnant women against malaria, WHO recommends intermittent preventive treatment (IPT) with sulphadoxine-pyrimethamine (SP) in stable transmission areas. Unfortunately, rates of SP resistance are now quite high in most of the world. For example, *Plasmodium falciparum *resistance to SP is greater than 57% in the eastern part of the Democratic Republic of Congo (DRC) [8-10]. The growing threat of SP resistance has resulted in substantial concern that SP may become ineffective for IPT in the very near future. Therefore, there is an urgent need to identify efficacious alternative drug regimens [11,12].

Artemisinin-based combination therapy (ACT) is the current first-line treatment for acute malaria in most malaria-endemic countries [1]. Currently, recommended ACT dosage regimens are based on studies conducted in non-pregnant patients; however, the pharmacokinetics of many drugs is altered during pregnancy. WHO endorses the use of ACT for patients in the second and third trimesters of pregnancy with acute uncomplicated falciparum malaria [13], but specific dosing recommendations for pregnant women have not been developed due to insufficient pharmacokinetic data in this population. There are also safety concerns regarding the use of artemisinin derivatives in pregnancy, particularly in the first trimester. In response to evidence from animal studies of artemisinin-associated embryotoxicity [14], WHO guidelines constrain the use of these derivatives in the first trimester to cases of therapeutic failure with alterative agents [13].

Additional pharmacokinetic and safety information regarding the use of artemisinin derivatives in pregnancy are needed [15-18]. To date, no pharmacokinetic study for an artemisinin derivative has been conducted that included postpartum or non-pregnant controls. Additionally, no such pharmacokinetic study has been conducted with asymptomatic parasitaemic women, the candidate population for IPT. This paper reports the results of a safety and pharmacokinetic study of a single oral dose of artesunate (AS) given to asymptomatic but parasitaemic pregnant women with *P. falciparum *infection, the same women three months postpartum, and a parallel parasitaemic non-pregnant control group.

## Methods

### Study population

The study was conducted at Maternité des Soeurs de Saint Marc de Kingasani, the largest maternity service in Kinshasa. All women from 18 to 40 years of age presenting for antenatal care at less than 22 weeks of gestation assessed on last menstrual period (LMP), were screened for eligibility. Gestational age (GA) was confirmed by ultrasound using the Hadlock method [19]. Women with gestational ages between 8 and 21 weeks (inclusive) were invited to participate in the study. Women were asked during to return during the second trimester of pregnancy (22-26 weeks GA); those not enrolled during the second trimester were screened again during the third trimester (32-36 weeks). Eligibility for the study was established during the women's return visits. Eligibility criteria included the following: no fever (temperature < 37.5°C), infection with *P. falciparum *(parasite count between 200 to < 300,000 parasites/μL), haematocrit ≥30%, HIV seronegativity, and the absence of other major medical problems (e.g. diabetes, chronic hypertension, etc.).

To determine if women met the eligibility criteria, a complete medical history and physical examination was performed by a study physician; sociodemographic data was collected by a study nurse. Haematocrit was measured using a microhaematocrit centrifuge (Hawksley Haematospin 1400, Hawksley & Sons, Ltd, UK). Thick and thin blood films were stained with Giemsa and then read to determine parasite species and the density of *P. falciparum *asexual stages. Parasite density, based on examination of at least 200 high-power microscope fields, was calculated as (# trophozoites/#white blood cells)*6000. Human Immunodeficiency Virus (HIV) serology was determined using the Determine 1/2 HIV Test Kit (Inverness Medical Professional Diagnostics, Princeton, NJ). Liver function was assessed by measuring aspartate aminotransferase (AST) and alanine aminotransferase (ALT) in plasmaPiccolo Portable Chemistry Analyser, Abaxis, USA). Alpha-1-Acid glycoprotein was determined in serum samples using radial immunodiffusion (Kent Laboratories, Bellingham, WA, USA).

There were two groups of non-pregnant controls. One group of controls (n = 26) was comprised of the pregnant study subjects at their three month postpartum visits. The second group of controls (n = 25) consisted of malaria-infected, asymptomatic non-pregnant women. Women were recruited from the area around the hospital and from one suburban health center. Interested women were directed to the research center where they were tested to assess eligibility using the same criteria as the pregnant cases.

In order to confirm malaria infection, DNA was later extracted from dried blood spots and analysed by real time PCR using pan-species primers and probe [20].

### Ethical clearance and consent

The study protocol was approved by the ethics committees of Kinshasa School of Public Health, the University of North Carolina at Chapel Hill, and RTI International. The study was first introduced to the pregnant women during their first antenatal clinic (ANC) visit in order to obtain consent for early ultrasound screening. Consent for laboratory screening was obtained from these women when they returned during their second or third trimester of pregnancy, and from the non-pregnant controls at the time of enrollment. Consent for the two days inpatient was obtained from all subjects after determining eligibility.

### Treatment and sampling

Study subjects received an oral dose of 200 mg AS as four 50 mg tablets (manufactured by Guilin Pharmaceutical Co. Ltd, Guangxi, Republic of China) after a four-hour fasting period. The four tablets were given orally with 240 mL water under medical supervision. A catheter was inserted into a forearm vein for pharmacokinetic sample collection and flushed after each blood draw with saline solution. Blood (5 ml) was drawn from the IV catheter within a 30 minute period before drug intake and then at the following times after AS intake: 0.25, 0.5, 0.75, 1, 1.5 2, 3, 4, 6, and 8 hours. Sodium fluoride/potassium oxalate was used as an anticoagulant. Blood for malaria smears were taken by finger prick at pre-dose and at 12, 24, 30, 36, 42 and 48 hours post-dose. Malaria smears were also performed on days 7, 14 and 28.

Blood samples were centrifuged immediately at -4°C (1,100 × g/or 2600 rpm, 10 min; Eppendorf refrigerated centrifuge) and the plasma initially stored in liquid nitrogen before being transferred to a -80°C freezer. Twenty-four hours after receiving AS, malaria-infected women received 1725 mg SP to complete malaria treatment in accordance with National Malaria Control Program guidelines. The persistence of malaria infection and adverse events were tracked for 28 days after SP administration. Babies born from the pregnant women cohort were followed until one year of age and development assessed.

### Drug assay

Plasma concentrations of AS and its active metabolite dihydroartemisinin (DHA) were determined using a validated liquid chromatography-mass spectrometric method described by Naik *et al *[21] with slight modifications. Briefly, AS, DHA and the internal standard artemisinin were extracted from 0.25 mL of human plasma using solid phase extraction. The reconstituted extracts were chromatographed isocratically and the compounds detected and quantified by mass spectroscopy. The lower limit of quantification for both AS and DHA was 1 ng/mL. Unbound DHA was assayed using ultrafiltration.

### Pharmacokinetic and statistical analysis

Plasma AS and DHA concentration data were analyzed by noncompartmental methods using the program WinNonlin, version 5.2 (Pharsight, Mountain View, CA). Specifically, C_max _and T_max _were taken from observed concentrations and the terminal slope, λ_z_, determined for DHA by non-linear regression of the terminal portion of the concentration vs. time profile using a 1/Y weighting. The DHA elimination half-life was calculated as 0.693/λ_z_. The area under the DHA concentration-time curve (AUC_total_) was determined using the trapezoidal method and total DHA concentrations, with extrapolation of the AUC after the final concentration estimated as C_last_/λ_z_. The apparent plasma clearance (CL/F) was determined as Dose/AUC_total _and apparent volume of distribution (Vd/F) calculated as Dose/(λ_z _* AUC_total_) where the AS dose was assumed to be totally and exclusively converted to DHA. Thus the DHA dose was assumed to be equal to the AS dose on a molar basis (DHA dose = AS dose * (284.35/384.42)), the latter ratio representing the molecular weights of DHA and AS. The DHA free fraction (FF) was determined as the ratio of unbound/total drug concentration at each time point where both measurements were collected, with mean FF calculated for each participant within a pharmacokinetic visit. The area under the unbound DHA concentration-time curve (AUC_free_) was calculated as FF*AUC_total_. The AUC of AS was also determined using the trapezoidal method. Concentrations below the lower limit of quantification were assigned a value of zero. The terminal slope of the AS concentration profile could not be estimated due to the limited samples after C_max _with concentrations above the lower limit of quantification.

The primary pharmacokinetic outcome measured to assess dosing adequacy was DHA AUC_free_. The AUCs for unbound DHA during pregnancy and postpartum were compared using each subject as her own control. The ratio of the AUC during pregnancy to the AUC at postpartum (after log transformation) was considered a measure of the impact of pregnancy on systemic exposure. The interval between population ratios of 0.66 and 1.5 was defined as an interval of "no effect" of pregnancy on pharmacokinetics. The "no effect" interval was selected based on the dose-ranging study by Angus and coworkers [22]. Their sigmoid E_max _pharmacodynamic model suggested that the dose with 50% maximal effect on parasite clearance time (PCT) was 1.46 mg/kg or ~100 mg, with a sharp increase in response around this value (Hill slope coefficient of 10). Based on these data and available formulation strengths, we chose an exposure of 66% for non-pregnant women (equivalent to 132 mg) as the lower bound of our no-effect window. If the 90% confidence interval (CI) of pregnancy/postpartum DHA AUC_free _was determined to be within the 0.66 and 1.5 interval, then the DHA exposure was deemed as clinically equivalent between pregnancy and postpartum. Statistical analyses were performed using SAS/STAT software, version 9.2 (SAS Institute, Inc., Cary, NC, 2008). For comparisons involving non-compartmental parameters, p-values provided with geometric mean estimates and 90% CIs were based on (paired or 2-sample) t-tests (on logged data, except as noted) and with medians were based on (signed rank or rank sum) Wilcoxon tests. Spearman correlations were used to look for associations among the pregnant and control population. All p-values were 2-sided.

## Results

### Patients

Between May 2007 and November 2008, 13 pregnant women were enrolled in their second trimester and 13 in their third trimester. Twenty-five non-pregnant women with asymptomatic falciparum malaria were also enrolled. The demographic and medical characteristics of the three groups are shown in Table 1. There were no significant differences among any of the three groups in age, height, or education level. All women had normal temperatures, normal blood pressures, a haematocrit ≥ 30%, negative HIV tests result, normal physical exams and no histories of chronic illness or of drug intake (other than antipyretics). Pregnant women had a significantly lower haematocrit and higher parity.

**Table 1:** Demographic characteristics on admission of women with *Plasmodium falciparum *infection (pregnant cases and non-pregnant controls)

	Pregnant cases					
			
Characteristic	Window 1 (22-26 wks GA)	Window 2 (32-36 wks GA)	P-value*	All	Non-pregnant controls	P-value†
N	13	13		26	25	

Age (years)‡	21.7 ± 2.7	26.6 ± 4.4	0.0065	24.2 ± 4.4	24.8 ± 5.5	0.79

Height (m)‡	1.6 ± 0.1	1.6 ± 0.1	0.56	1.6 ± 0.1	1.6 ± 0.1	0.77

Weight (kg)‡	55.5 ± 8.3	63.0 ± 7.2	--	--	55.2 ± 10.6	--
		
Postpartum (3 months) weight (kg)‡	53.7 ± 7.6	58.0 ± 4.3	0.14	55.8 ± 6.4		0.29

BMI (kg/m^2^)‡	21.2 ± 2.8	24.4 ± 2.2	--	22.7 ± 3.0	21.0 ± 3.2	0.055
		
Postpartum (3 months) BMI (kg/m^2^)‡	20.3 ± 2.3	22.6 ± 1.7	0.017	21.4 ± 2.3		0.29

Education (years)‡	9.0 ± 3.6	9.5 ± 3.0	0.57	9.2 ± 3.3	9.8 ± 3.8	0.73

Parity§	0 (0 - 2)	1 (0 - 4)	0.081	1 (0 - 4)	0 (0 - 4)	0.0013

Parity			0.23			0.0002
				
0	7 (53.8)	4 (30.8)		11 (42.3)	23 (92.0)	
				
1	5 (38.5)	3 (23.1)		8 (30.8)	0 (0.0)	
				
2	1 (7.7)	4 (30.8)		5 (19.2)	1 (4.0)	
				
3-4	0 (0.0)	2 (15.4)		2 (7.7)	1 (4.0)	

GA at ultrasound screening (weeks)‡	18.9 ± 1.5	18.5 ± 1.5	0.41	--	--	--

GA at study screening (weeks)‡	22.4 ± 0.8	33.2 ± 1.4	--	--	--	--

Body temperature (°C)║	36.5 (36.1 - 36.7)	36.4 (36.2 - 36.5)	0.66	36.5 (36.1 - 36.6)	36.6 (36.4 - 36.8)	0.044

Haematocrit (%)║	30.0 (30.0 - 30.0)	32.0 (31.0 - 33.0)	0.035	30.5 (30.0 - 33.0)	38.0 (34.0 - 38.0)	<.0001

Dosage (mg/kg) ║	3.45 (3.17-4.17)	3.08 (2.99-3.17)	0.017	3.17 (3.08-4.00)	3.86 (3.39-4.17)	0.061

* Pregnant cases, Window 1 vs Window 2; Fisher's exact and Wilcoxon rank sum tests.

† All pregnant cases vs. nonpregnant controls; Fisher's exact and Wilcoxon rank sum tests.

‡ Values given as mean ± standard deviation.

§ Values given as median (range).

║ Values given as median (interquartile range).

The median parasite densities at screening were 528 parasites/μL of blood (interquartile range: 372 - 842) in the pregnant group and 807 parasites/μL (interquartile range: 325 - 2215) in the non-pregnant group; these were not significantly different (Table 2). All were *P. falciparum *monoinfections, except for one pregnant case with a mixed infection (*P. falciparum *and *Plasmodium malariae*). Antenatal women in Window 1 received slightly higher doses on a mg/kg dosage than those in Window 2, but there were no significant differences between the pregnant women and their controls.

**Table 2:** Pharmacodynamics of AS in malaria-infected women

	Pregnant cases				
			
Time point	Window 1 (22-26 wks GA)	Window 2 (32-36 wks GA)	All	Non-pregnant controls	P-value*
N	13	13	26	25	

**Screening**					

Parasitaemic - n (%)	13 (100.0)	13 (100.0)	26 (100.0)	25 (100.0)	--

Density (where > 0)†	635 (264 - 842)	516. (404 - 659)	528 (372 - 842)	807 (325 - 2215)	0.21

**0 hours (AS dosing)**					

Parasitaemic - n (%)	13 (100.0)	11 (84.6)	24 (92.3)	14 (56.0)	0.0029

Density (where > 0)†	414 (179 - 857)	183 (130 - 788)	247 (151, 828)	371 (124 - 957)	0.66

**12 hours**					

Parasitaemic - n (%)	3 (23.1)	0 (0)	3 (11.5)	1 (4.0)	0.61

Density (where > 0)†	87 (44 - 286)	-- --	87 (44 - 286)	24 --	0.44

* All pregnant cases vs. nonpregnant controls; Pearson chi-square, Fisher's exact, and Wilcoxon rank sum tests.

† Values given as median (interquartile range).

### Pharmacodynamics

Parasitaemia was confirmed in all subjects by PCR at the time of recruitment. However, two pregnant women and 11 non-pregnant controls were microscopically aparasitaemic by the time the pharmacokinetic experiment started, usually a day later. Of the 24 pregnant women who were parasitaemic at t = 0, 21 (87.5%) were aparasitaemic at 12 hrs (Table 2). Similarly, 13 of the 14 (93%) non-pregnant controls with parasites at t = 0 were aparasitaemic at 12 hours. One pregnant woman was parasitaemic on day 28. Two others had gametocytes on day 7, one of whom also had gametocytes on day 14. Two other women from the pregnant group were parasitaemic at three months postpartum; one of these had a mixed infection with (*P. falciparum *and *P. malariae*). There were no significant differences between pregnant cases and controls in the percentage of women who were non-parasitaemic at 12 hours (Table 2).

### Pharmacokinetics

In all three groups, AS was rapidly hydrolyzed to DHA, with AS concentrations below the lower limit of quantification (1 ng/mL) after 4 hours in all but two samples. The median AS AUCs were 205, 132 and 187 ng*h/mL for pregnancy, postpartum and non-pregnant women, respectively, with AUCs ranging among the subjects by more than 17-fold.

**Figure 1:**
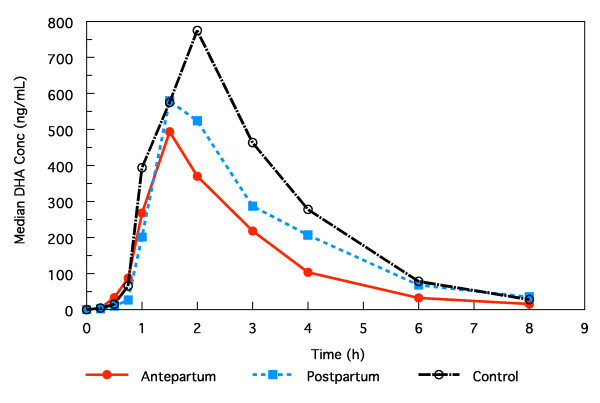
**Median plasma concentration of DHA in pregnant, postpartum, and non-pregnant subjects**.

The median plasma concentration profiles of DHA in the three groups are shown in Figure 1. The pharmacokinetic parameter estimates for DHA are summarized in Table 3. The pregnancy:postpartum, pregnancy:control, and postpartum:control ratios of geometric means for those parameters are shown in Table 4. Most DHA pharmacokinetic parameters were similar during pregnancy and postpartum after accounting for the higher free fraction during pregnancy. The estimated geometric mean and 90% CI for the ratio of the within-subject AUCs for free DHA for the women during pregnancy compared with three months postpartum was 0.99 (0.85, 1.15).

**Table 3:** DHA noncompartmental pharmacokinetic data in pregnant, postpartum, and non-pregnant women with *Plasmodium falciparum *infection.

	Pregnant cases			P-value		
			
Parameters	Ante-partum	Post-partum (3 months)	Non-pregnant controls	Ante vs. Post*	**Ante vs. Non-pregnant controls**†	**Post vs. Non-pregnant controls**†
C_max _(ng/mL)	904.6 [363.9 - 2541.9]	979.5 [414.5 - 2246.3]	1284.7 [539.8 - 3259.4]	0.89	0.24	0.090

T_max _(h)	1.50 [0.25 - 6.00]	1.50 [0.50 - 4.00]	1.52 [0.50 - 4.03]	0.95	0.57	0.34

AUC_all _(ng*h/mL)	1730.5 [1034.7 - 4085.5]	2122.2 [1308.9 - 4054.9]	2787.0 [1244.8 - 6363.5]	0.0080	0.0018	0.021

AUC_free _(ng*h/mL)	398.5 [138.7 - 663.9]	373.0 [186.5 - 708.3]	504.6 [197.9 - 1301.3]	0.80	0.057	0.043

CL/F (L/h)	85.5 [36.2 - 142.9]	69.7 [36.5 - 113.0]	53.1 [23.2 - 118.8]	0.0035	0.0018	0.021

CL/F (L/kg/h)	1.39 [0.75 - 2.46]	1.26 [0.63 - 2.32]	1.07 [0.53 - 1.89]	0.033	0.0025	0.011

T½ (h)	1.28 [0.83 - 2.71]	1.63 [0.92 - 2.18]	1.41 [0.86 - 6.55]	0.021	0.28	0.27

Vd/F (L)	157.4 [59.9 - 335.9]	158.3 [66.0 - 332.7]	128.2 [38.5 - 400.1]	0.82	0.098	0.071

Vd/F (L/kg)	2.84 [1.25 - 5.33]	3.00 [1.55 - 5.37]	2.45 [0.78 - 6.56]	0.54	0.17	0.083

FF	0.21 [0.13 - 0.31]	0.19 [0.11 - 0.27]	0.18 [0.09 - 0.39]	0.014	0.031	0.82

Medians [range] and nonparametric comparison of groups

* Wilcoxon signed rank test.

† Wilcoxon rank sum test.

**Table 4:** DHA noncompartmental pharmacokinetic data in pregnant, postpartum, and non-pregnant women with *Plasmodium falciparum *infection.

	Antepartum vs. Postpartum*		Antepartum vs. Non-pregnant controls†		Postpartum vs. Non-pregnant controls†	
	
Parameters	Estimate (90% CI)	P-value	Estimate (90% CI)	P-value	Estimate (90% CI)	P-value
C_max _(ng/mL)	1.06 (0.87, 1.30)	0.62	0.81 (0.63, 1.03)	0.15	0.77 (0.61, 0.98)	0.070

T_max _(h)	0.99 (0.74, 1.33)	0.95	0.90 (0.66, 1.23)	0.58	0.89 (0.67, 1.17)	0.47

AUC_all _(ng*h/mL)	0.87 (0.77, 0.98)	0.064	0.68 (0.57, 0.81)	0.0004	0.78 (0.66, 0.92)	0.014

AUC_free _(ng*h/mL)	0.99 (0.85, 1.15)	0.92	0.78 (0.63, 0.95)	0.039	0.78 (0.65, 0.95)	0.038

CL/F (L/h)	1.21 (1.09, 1.33)‡	0.0075	1.48 (1.24, 1.76)	0.0004	1.29 (1.09, 1.52)	0.014

CL/F (L/kg/h)	1.16 (1.03, 1.29)‡	0.040	1.36 (1.18, 1.57)	0.0008	1.24 (1.07, 1.44)	0.016

T½ (h)	0.84 (0.75, 0.96)	0.028	0.86 (0.72, 1.02)	0.15	1.03 (0.86, 1.22)	0.80

Vd/F (L)	0.97 (0.83, 1.14)	0.74	1.26 (1.00, 1.60)	0.097	1.32 (1.04, 1.67)	0.056

Vd/F (L/kg)	0.95 (0.81, 1.12)	0.61	1.18 (0.96, 1.44)	0.19	1.26 (1.02, 1.56)	0.073

FF	1.14 (1.05, 1.24)	0.013	1.15 (1.02, 1.28)	0.051	1.01 (0.89, 1.14)	0.92

Relative estimates (90% CIs) and parametric tests for group comparisons

* Geometric mean antepartum:postpartum ratio and 90% CI; 1-sample t-test on logged ratios.

† Ratio of geometric means and 90% CI; 2-sample t-test on logged data

‡ Mean antepartum:postpartum ratio and 90% CI; 1-sample t-test on ratio

The median DHA AUC was significantly higher in the non-pregnant control group than in the pregnant group (2787 vs 1731 ng*h/mL, respectively p = 0.0018; estimated ratio of geometric means, 0.68, 90% CI [0.57, 0.81], p = 0.0004); the difference in C_max _did not reach statistical significance (1285 vs 905 ng*h/mL respectively, p = 0.24; 0.81 [0.63, 1.03], p = 0.15). The estimated ratio of geometric means for free DHA AUC in the pregnancy and control groups was 0.78, 90% CI [0.63, 0.95], p = 0.039. The DHA free fraction was significantly higher in the pregnancy group than control groups but the free DHA AUC remained higher in non-pregnant than the pregnancy group.

**Figure 2:**
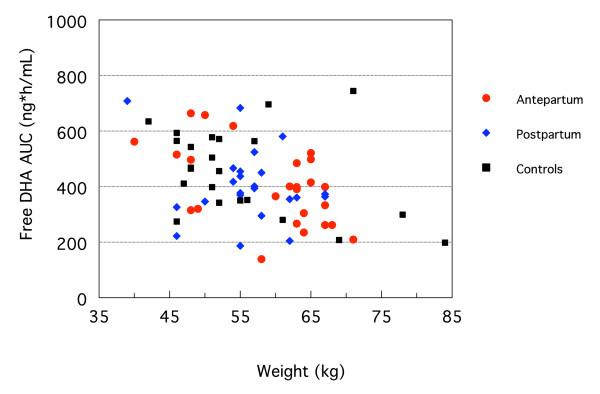
**Unbound DHA AUC versus body weight, by subject group**.

The relationship between free DHA AUC and body weight is shown in Figure 2. Larger women had lower AUCs in the malaria infected groups as weight was inversely correlated with AUC (r^2 ^= 0.38, p = 0.001). Albumin and AGP levels were lower in pregnant women than in postpartum or non-pregnant controls (p = 0.0003 and 0.0038, respectively, Additional file 1). These lower binding protein concentrations are likely responsible for the modest difference in the unbound DHA fraction.

### Safety

All pregnancies were followed until delivery and resulted in singleton live births. There were no congenital abnormalities. The median birth weight was 3025 grams [range 2130, 3620] with 7.7% (2/26) weighing ≤ 2500 grams. Twenty-three of the 26 babies were followed until one year of age and at that time had normal physical and neurological development.

There were no adverse events observed in any subjects. Statistically significant increases in some laboratory parameters were observed (Additional file 1), but the magnitude of these changes was small. In some subjects, transient elevations of ALT and AST above the upper limits of normal were observed. However, as these elevations were less than twice the upper limits of normal, they were considered clinically insignificant.

## Discussion

The study was conducted in the DRC, where high *in vivo *resistance to SP [8-10] could soon compromise the efficacy of SP for IPT in pregnancy. An IPT regimen incorporating an artemisinin derivative, such as AS, could provide a promising alternative to SP. However, despite the broad implementation of ACTs globally for treatment of acute falciparum malaria, questions remain regarding the safety, pharmacokinetics, and efficacy of artemisinin derivatives in pregnant women. The present study was designed to assess the safety and pharmacokinetics of AS in a population of asymptomatic parasitaemic women in the second and third trimesters of pregnancy, compared to the same women at three months postpartum and a control group of parasitaemic non-pregnant controls.

The pharmacokinetic analysis in this study indicates that in all three groups of women, AS was rapidly hydrolysed to its active metabolite DHA, becoming undetectable within 4 hours. This observed rapid AS disappearance is consistent with results previously reported in the literature [23,24]. This rapid disappearance precluded a comprehensive characterization of AS pharmacokinetics. However, given that DHA is the principle source of antimalarial activity following AS administration, the central focus of this pharmacokinetic analysis was the assessment of DHA pharmacokinetic alterations occurring during pregnancy. Pregnancy has been associated with substantial changes in the absorption, distribution, metabolism and excretion of a variety of drugs. High levels of progesterone may affect drug absorption. Increased plasma volume and altered drug binding protein concentrations can result in an increased volume of distribution. Pregnancy may increase metabolism of drugs, possibly through increased hepatic blood flow and increased free-fraction of drugs [25,26]. Decreased plasma concentrations of therapeutic drugs in pregnancy can be a result of increased volume of distribution, increased pre-systemic clearance, increased metabolism or excretion, decreased protein binding, or any combination of these factors. Given the importance of maintaining therapeutic anti-malarial blood levels, reductions in DHA blood levels resulting from such pregnancy-induced pharmacokinetic changes could potentially worsen clinical outcomes.

In this study, pregnant women had significantly lower plasma concentrations (AUC) of DHA s compared to non-pregnant controls. The 90% CI for DHA C_max_, AUC_all _and AUC_free _ratios in pregnancy compared to three months postpartum fell within the protocol specified 0.66-1.50 interval for the ratio. However, the median total and free AUCs for DHA in pregnant women were only 62% and 79% of the median total and free AUC for DHA in the non-pregnant controls. This decreased DHA exposure in pregnant women relative to non-pregnant controls is consistent with increased CL/F and possibly increased Vd/F during pregnancy.

There are several possible explanations for the apparent paradox of the similarity between DHA levels in pregnant women compared to levels in these women at three months postpartum but reduced levels compared to non-pregnant women controls. First, there are a variety of physiological and genetic factors that affect drug absorption, distribution and metabolism. A comparison of pharmacokinetic parameters in the same women during and after pregnancy mitigates the effect of most these potential confounders. Second, it is possible that many of the physiological changes that occur during pregnancy remain three months postpartum and continue to differentiate postpartum women from non-pregnant women. Only a few of the postpartum women were parasitaemic at the time of their repeat evaluation, which may also impact DHA pharmacokinetics. Finally, all women were lactating at three months post-partum; it is possible that lactation effects drug disposition.

Although the 90% confidence intervals for the DHA pharmacokinetic parameter ratios for the pregnant and postpartum subjects were within the 0.66-1.50 equivalence interval, the median values of AUC_all _and CL/F, but not Vd/F, in the postpartum subjects were trending toward those in the non-pregnant parallel-group subjects. Therefore, if the non-pregnant control group pharmacokinetic parameter estimates are representative of the pre-pregnant pharmacokinetics of the pregnant/post-partum group, then the return of pharmacokinetic parameters to presumed baseline may not have occurred completely at three months postpartum.

There are few previous pharmacokinetic studies in which postpartum subjects were compared intrasubject to the pregnant state. In a study of caffeine during and after pregnancy, the pharmacokinetics returned to predicted values in non-pregnant women within 4 days postpartum [27]. In another study, the estimated mean midazolam pharmacokinetic parameters, which reflected significantly decreased AUC and Cmax corresponding with increased apparent clearance during pregnancy, had returned to apparent normal in the postpartum period (weeks 6-10). In the same study, digoxin (p-glycoprotein substrate) pharmacokinetic parameters had also returned to normal by the post-partum period [28]. A multi-site, multi-country study on the pharmacokinetics of sulphadoxine and pyrimethamine during pregnancy and the postpartum period showed variable results by site and an inconsistency with pharmacokinetic parameters previously reported in the literature [29]. Lactation state has also been shown to modify alcohol pharmacokinetics in women [30]. Based on these examples, the time course to return to apparent "baseline" may, among other explanations, be chemotype-specific, lactation-state dependent, or pharmacogenomically-influenced. That there were inconsistencies reported for sulfadoxine-pyrimethamine both within- and between studies suggests that caution should be exercised in making dosing decisions based on a single study.

The presence of parasitaemia could potentially be contributing to the difference between the postpartum women and the non-pregnant controls, given that malaria infection itself may lead to altered pharmacokinetics [31]. Although all subjects in the present study, including postpartum subjects, were PCR-confirmed parasitaemic at screening prior to treatment with AS, only two of the postpartum women were parasitaemic by microscopy of Giemsa-stained blood films at the time of their pharmacokinetic assessments. Whether differences in parasitaemia influenced differences in pharmacokinetic parameter estimates is unknown. However, subjects were asymptomatic with low levels of parasites, which suggests that the influence of parasitaemia on pharmacokinetic parameter estimates may be minimal.

A prior study conducted by McGready *et al *in Thailand suggested that pregnancy was associated with decreased exposure to DHA, and increased DHA CL/F and Vd/F, as compared to results obtained in previous studies with non-pregnant adults [16]. This study evaluated the pharmacokinetics of DHA following AS administration using samples from the final day of a three day course of treatment for acute uncomplicated falciparum malaria in second and third trimester pregnant women. Their reported median value for DHA CL/F is higher than the values determined here, as well as higher than previously reported by Newton *et al *in non-pregnant patients with falciparum malaria during the acute or convalescent phase of infection [32]. The median DHA Vd/F observed in the McGready study (3.4 L/kg; 90% range 0.9-60.7) was also somewhat higher than the value observed in our analysis. This difference could, in part, be due to the fact that the women in the McGready study were given a different dosage of artesunate (4 mg/kg) in conjunction with Malarone^®^. However, the substantially greater degree of variability in DHA CL/F and Vd/F observed in the McGready study as compared to our study, as well as a lack of a postpartum or non-pregnant control group, complicates a direct comparison of findings.

Both the pregnant women and non-pregnant controls in our study displayed rapid clearance of parasitaemia following administration of the 200 mg oral dose of AS. Differences in parasite clearance were not detected between the groups, despite the lower DHA AUC of the pregnant women. However, the possibility that a higher dose of AS in pregnancy, especially in larger women, would enhance the parasite clearance cannot be excluded.

No apparent AS toxicity to either mother or child was observed in this study. Concerns about the safety of artemisinin derivatives in pregnancy indicate that toxicity may be confined to the first trimester, with studies in rodents and primates suggesting that the artemisinin derivatives may be embryotoxic [14]. The positive safety findings of this study provide additional evidence that any toxicity risk of first trimester use may not extend into later periods of gestation.

## Conclusions

Oral AS was found to be effective and without adverse effects in this study of Congolese pregnant women. No clinically relevant differences were seen in DHA pharmacokinetics between treated pregnant women and the same women three months postpartum. In contrast, the pregnant women had much lower AUCs than non-pregnant controls. These results suggest that further studies on the pharmacokinetics of AS in pregnant women are needed.

## Competing interests

The authors declare that they have no competing interests.

## Authors' contributions

JHF, MK, EC, RWR and RSM contributed to designing the study protocol. MD, MO, and JA wrote and produced clinical SOPs'. VL, JA and MO carried out ultrasound exams, biological sample and data collection. MK, EC, LF and MO carried out data analysis. MO, AKT and SRM drafted the initial manuscript. All the authors revised, read and approved the final manuscript.

## Supplementary Material

Additional file 1 **Changes in biochemical markers following AS administration**. All biochemical marker values given as mean ± standard deviation. Click here for file

## References

[B1] World Health Organization World Malaria Report 2009 2009 Geneva, Switzerland: World Health Organization

[B2] Shulman CE Dorman EK Bulmer JN Malaria as a cause of severe anaemia in pregnancy Lancet 2002 360 494 10.1016/S0140-6736(02)09662-9 12241758

[B3] Menendez C Ordi J Ismail MR Ventura PJ Aponte JJ Kahigwa E Font F Alonso PL The impact of placental malaria on gestational age and birth weight J Infect Dis 2000 181 1740 1745 10.1086/315449 10823776

[B4] Brabin B Prinsen-Geerligs P Verhoeff F Kazembe P Anaemia prevention for reduction of mortality in mothers and children Trans R Soc Trop Med Hyg 2003 97 36 38 10.1016/S0035-9203(03)90014-9 12886802

[B5] Steketee RW Nahlen BL Parise ME Menendez C The burden of malaria in pregnancy in malaria-endemic areas Am J Trop Med Hyg 2001 64 1-2 Suppl 28 35 11425175 10.4269/ajtmh.2001.64.28

[B6] Menendez C D'Alessandro U ter Kuile FO Reducing the burden of malaria in pregnancy by preventive strategies Lancet Infect Dis 2007 7 126 135 10.1016/S1473-3099(07)70024-5 17251083

[B7] World Health Organization A strategic framework for malaria prevention and control during pregnancy in the African region 2004 Brazzaville: WHO Regional Office for Africa

[B8] Alker AP Kazadi WM Kutelemeni AK Bloland PB Tshefu AK Meshnick SR dhfr and dhps genotype and sulfadoxine-pyrimethamine treatment failure in children with falciparum malaria in the Democratic Republic of Congo Trop Med Int Health 2008 13 1384 1391 10.1111/j.1365-3156.2008.02150.x 19055622 PMC2765712

[B9] Swarthout TD van den Broek IV Kayembe G Montgomery J Pota H Roper C Artesunate + amodiaquine and artesunate + sulphadoxine-pyrimethamine for treatment of uncomplicated malaria in Democratic Republic of Congo: a clinical trial with determination of sulphadoxine and pyrimethamine-resistant haplotypes Trop Med Int Health 2006 11 1503 1511 10.1111/j.1365-3156.2006.01710.x 17002724

[B10] Cohuet S Bonnet M Van Herp M Van Overmeir C D'Alessandro U Guthmann JP Short report: molecular markers associated with *Plasmodium falciparum *resistance to sulfadoxine-pyrimethamine in the Democratic Republic of Congo Am J Trop Med Hyg 2006 75 152 154 16837723

[B11] Sevene E Gonzalez R Menendez C Current knowledge and challenges of antimalarial drugs for treatment and prevention in pregnancy Expert Opin Pharmacother 2010 11 1277 1293 10.1517/14656561003733599 20408744

[B12] Ward SA Sevene EJ Hastings IM Nosten F McGready R Antimalarial drugs and pregnancy: safety, pharmacokinetics, and pharmacovigilance Lancet Infect Dis 2007 7 136 144 10.1016/S1473-3099(07)70025-7 17251084

[B13] World Health Organization Guidelines for the treatment of malaria 2010 2 Geneva, Switzerland: World Health Organization

[B14] Clark RL Embryotoxicity of the artemisinin antimalarials and potential consequences for use in women in the first trimester Reprod Toxicol 2009 28 285 296 10.1016/j.reprotox.2009.05.002 19447170

[B15] McGready R Stepniewska K Lindegardh N Ashley EA La Y Singhasivanon P White NJ Nosten F The pharmacokinetics of artemether and lumefantrine in pregnant women with uncomplicated falciparum malaria Eur J Clin Pharmacol 2006 62 1021 1031 10.1007/s00228-006-0199-7 17053895

[B16] McGready R Stepniewska K Ward SA Cho T Gilveray G Looareesuwan S White NJ Nosten F Pharmacokinetics of dihydroartemisinin following oral artesunate treatment of pregnant women with acute uncomplicated falciparum malaria Eur J Clin Pharmacol 2006 62 367 371 10.1007/s00228-006-0118-y 16552504

[B17] Rijken MJ McGready R Boel ME Barends M Proux S Pimanpanarak M Singhasivanon P Nosten F Dihydroartemisinin-piperaquine rescue treatment of multidrug-resistant *Plasmodium falciparum *malaria in pregnancy: a preliminary report Am J Trop Med Hyg 2008 78 543 545 18385345

[B18] Meshnick SR Artemisinin: mechanisms of action, resistance and toxicity Int J Parasitol 2002 32 1655 1660 10.1016/S0020-7519(02)00194-7 12435450

[B19] Hadlock FP Deter RL Carpenter RJ Park SK Estimating fetal age: effect of head shape on BPD AJR Am J Roentgenol 1981 137 83 85 6787895 10.2214/ajr.137.1.83

[B20] Taylor SM Juliano JJ Trottman PA Griffin JB Landis SH Kitsa P Tshefu AK Meshnick SR High-throughput pooling and real-time PCR-based strategy for malaria detection J Clin Microbiol 2010 48 512 519 10.1128/JCM.01800-09 19940051 PMC2815636

[B21] Naik H Murry DJ Kirsch LE Fleckenstein L Development and validation of a high-performance liquid chromatography-mass spectroscopy assay for determination of artesunate and dihydroartemisinin in human plasma J Chromatogr B Analyt Technol Biomed Life Sci 2005 816 233 242 10.1016/j.jchromb.2004.11.042 15664355

[B22] Angus BJ Thaiaporn I Chanthapadith K Suputtamongkol Y White NJ Oral artesunate dose-response relationship in acute falciparum malaria Antimicrob Agents Chemother 2002 46 778 782 10.1128/AAC.46.3.778-782.2002 11850261 PMC127461

[B23] Binh TQ Ilett KF Batty KT Davis TM Hung NC Powell SM Thu LT Thien HV Phuong HL Phuong VD Oral bioavailability of dihydroartemisinin in Vietnamese volunteers and in patients with falciparum malaria Br J Clin Pharmacol 2001 51 541 546 10.1046/j.1365-2125.2001.01395.x 11422013 PMC2014487

[B24] Batty KT Thu LT Davis TM Ilett KF Mai TX Hung NC Tien NP Powell SM Thien HV Binh TQ A pharmacokinetic and pharmacodynamic study of intravenous vs oral artesunate in uncomplicated falciparum malaria Br J Clin Pharmacol 1998 45 123 129 10.1046/j.1365-2125.1998.00655.x 9491824 PMC1873351

[B25] Frederiksen MC Physiologic changes in pregnancy and their effect on drug disposition Semin Perinatol 2001 25 120 123 10.1053/sper.2001.24565 11453606

[B26] Soldin OP Mattison DR Sex differences in pharmacokinetics and pharmacodynamics Clin Pharmacokinet 2009 48 143 157 10.2165/00003088-200948030-00001 19385708 PMC3644551

[B27] Brazier JL Ritter J Berland M Khenfer D Faucon G Pharmacokinetics of caffeine during and after pregnancy Dev Pharmacol Ther 1983 6 315 322 6628163 10.1159/000457332

[B28] Hebert MF Easterling TR Kirby B Carr DB Buchanan ML Rutherford T Thummel KE Fishbein DP Unadkat JD Effects of pregnancy on CYP3A and P-glycoprotein activities as measured by disposition of midazolam and digoxin: a University of Washington specialized center of research study Clin Pharmacol Ther 2008 84 248 253 10.1038/clpt.2008.1 18288078

[B29] Nyunt MM Adam I Kayentao K van Dijk J Thuma P Mauff K Little F Cassam Y Guirou E Traore B Doumbo O Sullivan D Smith P Barnes KI Pharmacokinetics of sulfadoxine and pyrimethamine in intermittent preventive treatment of malaria in pregnancy Clin Pharmacol Ther 2010 87 226 234 10.1038/clpt.2009.177 19776738

[B30] Pepino MY Steinmeyer AL Mennella JA Lactational state modifies alcohol pharmacokinetics in women Alcohol Clin Exp Res 2007 31 909 918 10.1111/j.1530-0277.2007.00387.x 17433009 PMC2265592

[B31] Teja-Isavadharm P Watt G Eamsila C Jongsakul K Li Q Keeratithakul G Sirisopana N Luesutthiviboon L Brewer TG Kyle DE Comparative pharmacokinetics and effect kinetics of orally administered artesunate in healthy volunteers and patients with uncomplicated falciparum malaria Am J Trop Med Hyg 2001 65 717 721 11791963 10.4269/ajtmh.2001.65.717

[B32] Newton P Suputtamongkol Y Teja-Isavadharm P Pukrittayakamee S Navaratnam V Bates I White N Antimalarial bioavailability and disposition of artesunate in acute falciparum malaria Antimicrob Agents Chemother 2000 44 972 977 10.1128/AAC.44.4.972-977.2000 10722499 PMC89800

