# Supplementary material for: Pharmacokinetics and pharmacodynamics of artesunate and dihydroartemisinin following oral treatment in pregnant women with asymptomatic Plasmodium falciparum infections in Kinshasa DRC
Source: Malar J. 2011 Feb 28;10:49. doi: 10.1186/1475-2875-10-49 (PMC3056842; doi:10.1186/1475-2875-10-49)
Supplement: Additional file 1 — Changes in biochemical markers following AS administration. All biochemical marker values given as mean ± standard deviation. [file 1475-2875-10-49-S1.DOCX]

**Supplemental Table.** Changes in biochemical markers following AS administration. All biochemical marker values given as mean ± standard deviation.

|  | **Pregnant cases** | | | | | |  | | |
| --- | --- | --- | --- | --- | --- | --- | --- | --- | --- |
|  | **Antepartum** | | | **3 Months Postpartum** | | | **Non-pregnant Controls** | | |
| **Characteristic** | **Baseline** | **Post dosing** | **P-value*** | **Baseline** | **Post dosing** | **P-value*** | **Baseline** | **Post dosing** | **P-value*** |
| Albumin (g/dl) | 2.55 ± 0.32 | 2.50 ± 0.32 | 0.35 | 3.30 ± 0.66 | 3.29 ± 0.50 | 0.78 | 3.40 ± 0.27 | 3.16 ± 0.26 | 0.0003 |
| Creatinine (mg/dl) | 0.58 ± 0.11 | 0.64 ± 0.09 | 0.018 | 0.76 ± 0.24 | 0.94 ± 0.27 | <.0001 | 0.77 ± 0.24 | 0.87 ± 0.19 | 0.14 |
| ALT(U/L) | 15.5 ± 4.4 | 17.2 ± 3.6 | 0.019 | 22.8 ± 11.3 | 28.3 ± 14.6 | 0.020 | 20.2 ± 10.6 | 30.1 ± 29.7 | <.0001 |
| AST(U/L) | 27.8 ± 6.0 | 29.4 ± 5.7 | 0.059 | 29.3 ± 9.3 | 37.6 ± 12.1 | <.0001 | 33.4 ± 5.2 | 40.2 ± 14.6 | 0.0001 |
| AGP (mg/dl) | 75.3 ± 21.7 | 77.5 ± 19.6 | 0.67 | 87.5 ± 26.4 | 106.8 ± 27.9 | <.0001 | 106.1 ± 34.0 | 115.7 ± 29.2 | 0.0038 |

* Post dosing vs. baseline, Wilcoxon signed rank test.
